# How COVID-19 Affects Lung Transplantation: A Comprehensive Review

**DOI:** 10.3390/jcm11123513

**Published:** 2022-06-18

**Authors:** Jiri Vachtenheim, Rene Novysedlak, Monika Svorcova, Robert Lischke, Zuzana Strizova

**Affiliations:** 1 Prague Lung Transplant Program, 3rd Department of Surgery, First Faculty of Medicine, Charles University, University Hospital Motol, 150 06 Prague, Czech Republic; jiri.vachtenheim@fnmotol.cz (J.V.J.); rene.novysedlak@fnmotol.cz (R.N.); monika.svorcova@fnmotol.cz (M.S.); robert.lischke@fnmotol.cz (R.L.); 2 Department of Immunology, Second Faculty of Medicine, Charles University, University Hospital Motol, 150 06 Prague, Czech Republic

**Keywords:** lung transplantation, COVID-19, immunosuppression, treatment, transplant activity

## Abstract

Lung transplant (LuTx) recipients are at a higher risk of developing serious illnesses from COVID-19, and thus, we have closely reviewed the consequences of the COVID-19 pandemic on lung transplantation. In most transplant centers, the overall LuTx activity significantly declined and led to a specific period of restricting lung transplantation to urgent cases. Moreover, several transplant centers reported difficulties due to the shortage of ICU capacities. The fear of donor-derived transmission generated extensive screening programs. Nevertheless, reasonable concerns about the unnecessary losses of viable organs were also raised. The overall donor shortage resulted in increased waiting-list mortality, and COVID-19-associated ARDS became an indication of lung transplantation. The impact of specific immunosuppressive agents on the severity of COVID-19 varied. Corticosteroid discontinuation was not found to be beneficial for LuTx patients. Tacrolimus concentrations were reported to increase during the SARS-CoV-2 infection, and in combination with remdesivir, tacrolimus may clinically impact renal functions. Monoclonal antibodies were shown to reduce the risk of hospitalization in SOT recipients. However, understanding the pharmacological interactions between the anti-COVID-19 drugs and the immunosuppressive drugs requires further research.

## 1. Introduction

The global impact of the Coronavirus Disease 2019 (COVID-19) pandemic keeps disproportionally affecting the most vulnerable human subpopulations [1,2,3]. Among these subpopulations, patients receiving immunosuppressive treatments continue to pose serious challenges regarding the prevention of their severe or fatal outcomes of COVID-19 infection [3,4,5].

In solid organ transplantations (SOT), various unprecedented challenges, such as limited donor pool, detailed screening for severe acute respiratory syndrome coronavirus 2 (SARS-CoV-2) in both organ donors and recipients, as well as management of infected recipients, had to be faced [6,7,8].

Though COVID-19-associated mortality among SOT recipients kept rising, a substantial reduction in transplantation procedures was reported throughout various countries [9]. As expected, this phenomenon led to an increase of people on waiting lists, with ensuing consequences [10,11,12].

COVID-19 was demonstrated to predominantly affect the respiratory tract, and, therefore, lung transplantations may be more susceptible to the detrimental impact of the COVID-19 pandemic [5,13]. Due to an unclear COVID-19 status in lung donors, a significant proportion of possible donor lungs was declined [14,15]. On the other hand, COVID-19-associated acute respiratory distress syndrome (ARDS) has become a novel disease entity that was shown to benefit from lung transplantation [16].

In this review, we attempted to address the main challenges of lung transplantations in the era of COVID-19. We have closely collected all available data on transplant activity, precaution measures, and clinical management of both donors and recipients, as well as therapeutic management of the COVID-19 infection in lung transplant (LuTx) recipients.

## 2. Methods

**Figure 1:**
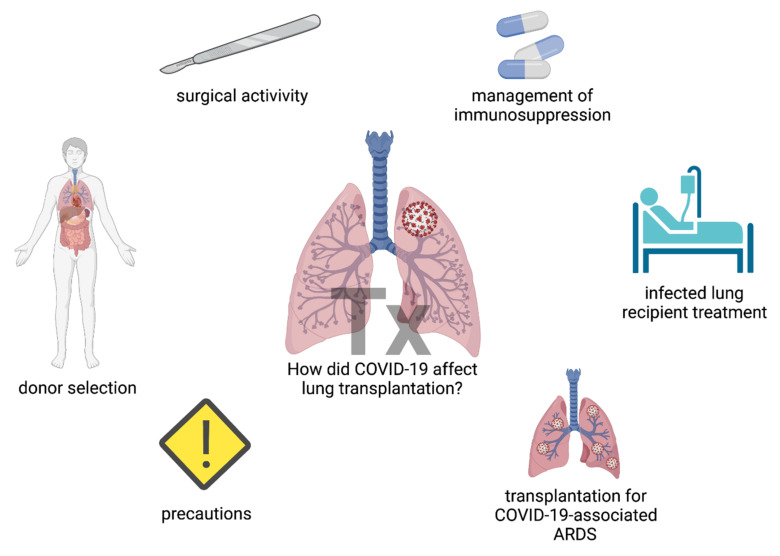
The areas of lung transplantation that were affected by the COVID-19 pandemic.

We conducted a comprehensive review of the literature on the impact of the COVID-19 pandemic on lung transplantation. COVID-19, lung transplantation, SARS-CoV-2, lung donor, and lung recipient, were used as the keywords in the search strategy. Only English-written and peer-reviewed reports published in indexed international journals until December 2021 were reviewed. Databases used for the search included Medline/Pubmed, Scopus, and Web of Science. The review outline is summarized in Figure 1.

Different aspects of lung transplantation, such as donor selection, surgical activity, and management of therapy, faced unprecedented challenges. Novel precaution measures were applied, and COVID-19-associated ARDS became an indication for lung transplantation. Figure 1 was created with BioRender.com (agreement no. IU23TYL40X).

## 3. Lung Transplant Activity during COVID-19 Pandemic

After COVID-19 was announced as a global pandemic in March 2020, the risk of COVID-19-associated deaths in SOT patients has become a major concern [17]. Even though the COVID-19 pandemic was shown to significantly decrease the total number of organ transplants in most countries, the overall transplant activity varied [9]. The geographic disparities in transplant activity were largely driven by the governmental regulations, lockdown policies, and recommendations of local transplant societies [18].

Coiffard et al. evaluated the transplant activity of 78 LuTx centers from 15 countries during the pandemic period [9]. In this international survey, only 19% of the centers reported stable LuTx activity, with no significant decrease during the COVID-19 pandemic [9]. Restriction of LuTx activity to urgent cases was observed in most of the centers (47%). In the United States, deaths on the LuTx waiting list rose by approximately 12%. A limited number of transplant centers performed LuTx for COVID-19-associated ARDS, but most centers agreed that COVID-19-infected patients with post-ARDS fibrosis will represent candidates for LuTx in the future [9].

An observational study by Aubert et al. investigated the impact of the COVID-19 pandemic on worldwide organ transplantation activity [19]. In this study, kidney transplantation was found to be the most affected area, followed by lung, liver, and heart transplantation. The worldwide transplant activity substantially decreased during the first three months of the pandemic. The losses, however, stabilized after June 2020 [19].

The transplant activity in the United States has been characterized by a significant regional disparity [20]. During the first three months of the COVID-19 pandemic, all LuTx centers continued with their regular activity. Nonetheless, this period was followed by a significant decrease in areas of high COVID-19 prevalence [20]. The donation services in the areas with high COVID-19 prevalence reported a decrease in organ availability. Waiting list activations decreased in 18 out of 22 transplant centers [20].

In a study by Johal et al., a 69.23% decline in the volume of organ transplantation was observed in Australia as of August 2020. The LuTx rates, however, increased when both social and travel restrictions were applied [21]. The causes of donor death were also affected by the COVID-19 pandemic, with suicide and overdose becoming 2.65 times more frequent [21].

A retrospective analysis of transplant activity across all adult and pediatric LuTx centers in the UK was provided by Hardmann et al. [22]. In the early pandemic period, the number of donors fell by 48%, with the most profound decrease in the number of donors after circulatory death (67%). Lung utilization from deceased donors was reduced to 10%, which was in contrast with the same period of 2019 where lung utilization was 22%. The overall LuTx activity significantly declined, with a reduction of 77%. Following other European countries, all UK transplant centers had a specific period of restricting lung transplantation to urgent cases [22].

A cross-sectional survey evaluating LuTx activity in Germany was provided by Michel et al. In this study, 50% of the LuTx centers reported difficulties during the pandemic era [23]. One of the reported difficulties included the shortage of intensive care unit (ICU) capacities. A total of 88% of the centers had no obstacles with the organ procurement procedures. Most centers required mandatory SARS-CoV-2 testing in recipients. The overall LuTx activity in Germany did not display any significant differences between the same period in 2019 and 2020 [23].

On the contrary, data from the Brazilian Transplantation Registry demonstrated a significant decline in transplant activity [24]. The pool of available organ donors decreased and affected mainly lung transplantations [24].

In a study by Kwapisz et al. evaluating the SOT activity in Poland, the number of organ donors dropped to 58% of its pre-pandemic value. Surprisingly, this phenomenon did not affect lung, heart, nor living donor liver transplants, due to less stringent donor acceptance criteria. However, all kidney transplantations and deceased donor liver transplantations were severely affected [25].

The unknown nature of COVID-19 infection and the possible impacts of the disease in immunosuppressed organ recipients affected several transplant societies, and led to the postponement of non-urgent transplantations. Furthermore, the possible risk of donor-to-recipient transmission caused a decrease in lung utilizations, thus, affecting the pool of available organ donors. Under those circumstances, the COVID-19 pandemic has put a lot of pressure on transplant centres all over the world.

## 4. Donor Selection, Precautions, and Lung Transplant Management during the COVID-19 Pandemic

The highly infectious nature of SARS-CoV-2 raised reasonable concerns about the donor-derived transmission, and thus, novel recommendations on both donor and recipient assessment were established [5].

Currently, lungs from donors with ongoing SARS-CoV-2 infection are not accepted for transplantation [26]. However, as the disease continues to spread, a significant proportion of donors are expected to have a COVID-19 exposure in the future [26,27]. Considering the current donor shortage and the waiting-list mortality, it is generally acknowledged that a history of resolved SARS-CoV-2 infection alone is not a contraindication to lung donation [27].

Donor-derived infection in both the recipient and the transplant surgeon was previously reported in donors who tested negative for SARS-CoV-2 by nasopharyngeal swab [15,28]. These donors were later found SARS-CoV-2-positive by real-time polymerase chain reaction (RT-PCR) from the bronchoalveolar lavage (BAL) fluid [15,28]. Thus, it is strongly recommended to provide SARS-CoV-2 screening in both the upper and lower respiratory tract to address the epidemiologic exposures of the donor [15,28,29,30,31].

Since accurate diagnostics are essential for the clinical treatment of infected individuals in the general population, RT-PCR assays may represent the most powerful diagnostic tool, as they have shown excellent analytical sensitivity and specificity from respiratory specimens [32]. However, false-negative RT-PCR results have also been observed. Such cases were attributed to non-exact specimen collection, testing in the early incubation phase of the disease, or specimen processing errors [32]. Querrey et al. demonstrated that even though respiratory tract presumably carries the highest viral burden of SARS-CoV-2, lung donation is still possible in donors with a recent history of COVID-19 infection. Moreover, repetitive RT-PCR testing from the lower respiratory tract and close evaluation of diverse aspects reflecting the probability of transmissible COVID-19 infection may decrease the likelihood of false positivity, and mitigate organ discard [27].

A chest computed tomography (CT) scan in donors may rule out the presence of residual signs of COVID-19-associated lung injury (ground-glass opacities, pulmonary fibrosis, or interstitial lung disease) [26].

High sensitivity of the chest CT for detecting COVID-19 (more than 95%) has been reported, and is, therefore, useful in the identification of cases with a false-negative PCR test [26,33]. However, the specificity of the chest CT for COVID-19 remains low [30]. Of note, high-resolution CT (HR-CT) was previously shown to play a critical role in early diagnosis of complications after lung transplantation, and this has also been proven during the COVID-19 pandemic in LuTx recipients [34,35].

Even though the COVID-19 pandemic has been among the toughest moments in recent transplant history, the rapid development of SARS-CoV-2 screening algorithms in both donors and recipients has minimized the possible detrimental impact of the situation. The appropriate selection of organ donors with minimal risk of viral transmission to the recipient has become a critical step during the COVID-19 pandemic, and this may, in the future, serve as a valuable prerequisite for the management of lung transplantations during other potential pandemics.

## 5. Lung Transplantation for SARS-CoV-2 Infection—Associated ARDS and Post-COVID Pulmonary Fibrosis

Despite the continually evolving management of SARS-CoV-2-infected patients, data demonstrate a progression to severe respiratory failure and ARDS in up to 42% of hospitalized patients, and the mortality rates of patients with COVID-19 requiring mechanical ventilation between 20% and 40% [36,37].

Lung transplantation is the well-established ultimate treatment for a variety of end-stage chronic lung diseases worldwide, but its role in patients with ARDS remains controversial. Currently, lung transplantation is an acceptable indication in emergency settings, and might be considered in selected patients with refractory ARDS [38,39,40,41].

To date, the evidence on lung transplantation even for non-covid ARDS is limited to a small number of case reports and a few larger case series [42]. However, these limited data may help navigate the treatment of ARDS following COVID-19 infection. Chang et al. reported single-center outcomes on nine patients who underwent lung transplantation for ARDS with a median survival time after the procedure of 64 months, and the 3-year survival rate for the recipients was 78% [43]. Frick et al. reported the post-transplant outcome of ARDS patients from three high-volume European lung transplant centers. The authors identified 13 cases of lung transplantation for ARDS over a period of 22 years, reporting a 30-day mortality of 7.7%. Moreover, 1- and 5-year survival rates were 71.6% and 54.2%, respectively [42]. These studies, along with the most recent retrospective analysis from 18 European lung transplant centers, suggest that salvage lung transplantation for ARDS might be a feasible approach with acceptable outcomes. Yet, such transplantation remains ethically and technically very challenging [42,43,44].

Extracorporeal membrane oxygenation (ECMO) experienced remarkable progress over recent years, and represents a crucial therapeutic approach for late-stage ARDS [45]. Lung injury and function can recover on ECMO within weeks to months [46]. However, some patients with ARDS still exhibit progression to end-stage lung disease, and thus, cannot be successfully separated from ECMO [38].

Currently, one of the most extensive reports by Bharat et al. evaluated twelve patients who underwent lung transplantation for COVID-19-associated ARDS at different institutions [39]. All twelve patients presented intraoperatively with dense pleuropulmonary adhesions, associated with highly vascularized and thickened mediastinal and parietal pleura [39]. The use of ECMO was required in all cases. The pre-operative ECMO bridge (11 from 12 patients, 91.7%) and intraoperative venoarterial extracorporeal membrane oxygenation (VA-ECMO) support led to a high intraoperative utilization of blood products, with a median of eight units of packed red blood cells (IQR 5–15) and four units of fresh frozen plasma (IQR 3–7) per person. Venovenous extracorporeal membrane oxygenation (VV-ECMO) was empirically continued after the transplantation in ten cases (83.3%), due to the high risk of primary graft dysfunction. As anticipated, the patients exhibited a difficult recovery. However, the 30-day survival rate was 100% in this unique cohort [39].

In accordance with the conclusions made by Bharat et al., Lepper et al. also discussed the success of the bridging of patients to lung transplantation with ECMO. However, the authors also raised several medical and ethical concerns of this approach, such as disadvantaging patients on the waiting list when high-urgency candidates with ARDS become acceptable LuTx recipients during the pandemic [47].

Another recent study by Lang et al. retrospectively evaluated 19 patients undergoing lung transplantation for COVID-ARDS. In this study, the 30-day mortality of the recipients was 0%, and 14 out of 19 patients were alive at the median follow-up of 134 (47–450) days [48]. Experts in the field suggested several factors that should be taken into consideration when assessing a patient with COVID-19-associated ARDS regarding potential candidacy for lung transplantation (based on initial experience) apart from the standard criteria for lung transplantation [38,39,49,50]. These factors are summarized in Table 1.

Post-covid pulmonary fibrosis is another disease entity associated with the abnormal healing of the injured lung parenchyma [52]. Patients with post-covid pulmonary fibrosis have also been identified to profit from lung transplantation, and thus, several studies already demonstrated the success of such an approach. Gogia et al. presented a case of a 34-year-old male who developed end-stage pulmonary fibrosis following COVID-19 infection. A bilateral lung transplantation was carried out in this patient, and they were allowed a discharge from the hospital on day 15 following lung transplantation [53]. In accordance with Gogia et al., Hall et al. also described a case of severe post-covid pulmonary fibrosis in a 52-year-old female. In this patient, bilateral lung transplantation led to a fast recovery, and proved the efficacy of this approach [54]. Mohammadi et al. also proposed that even though several treatment modalities are currently available for post-covid pulmonary fibrosis, lung transplantation may be the only life-saving treatment in selected cases [55].

Lung transplantation for covid-associated ARDS and/or post-covid pulmonary fibrosis currently represents an area with many aspects that remain to be elucidated. To address these aspects, more experience with these challenging transplantations is needed. The selection of the optimal candidate and the optimal timing of the surgery may require a highly individualized approach for each patient.

## 6. SARS-CoV-2-Infected Recipient—Management of Immunosuppression

Lung transplant recipients require long-term immunosuppression to prevent organ rejection and a subsequent loss of the lung allograft [56,57]. Recent studies demonstrated that some immunosuppressants, such as anti-IL-6 and other biological disease-modifying anti-rheumatic drugs (bDMARDs), may provide a certain level of protection against COVID-19, but the actual impact of anti-rejection immunosuppressive treatment on the COVID-19 disease severity is unclear [58,59,60].

Mycophenolic acid (MPA) restrains DNA synthesis, and thus, inhibits proliferation of the T and B cells, as well as the generation of immunoglobulins [61]. Several studies demonstrated that MPA inhibits SARS-CoV-2 in-vitro, but the inhibitory effect of MPA on the SARS-CoV-2 replication in-vivo was not confirmed so far [62,63]. General recommendations suggest tapering or maintaining the dose, particularly in patients with lymphopenia [64,65].

Calcineurin inhibitors (CNIs) inhibit the T-cell activation by decreasing the dephosphorylation of the nuclear factor of activated T cells (NFAT) [57]. In particular, tacrolimus and cyclosporine A, the two major CNIs, interfere with the pro-inflammatory processes, and, therefore, serve as a powerful therapeutic tool in the treatment of LuTx recipients [66]. Interestingly, CsA was found to block the SARS-CoV-2 replication and to prevent various infectious patterns of COVID-19 by interfering with the angiotensin II [67]. Another important fact is that CsA forms complexes with cyclophilins, which are essential for the replication of SARS-CoV-2 [57]. Lai et al. showed that both immunosuppressive drug types, CNIs and mycophenolic acid, could be used as a treatment strategy to reduce viral replication [63]. However, to achieve appropriate concentrations of CsA in infected tissues, three- to six-fold higher doses would be necessary to attain these effects [63]. As expected, these high doses would bear a significant risk of systemic toxicity [63].

To date, it is generally recommended to continue with the CNI therapy during COVID-19 infection [68,69]. Salerno et al. stated that SARS-CoV-2 infection increases tacrolimus concentration. Therefore, closer monitoring of tacrolimus concentration should be considered in SARS-CoV-2-positive LuTx patients [70]. Switching to cyclosporine A from tacrolimus is not encouraged [70].

Corticosteroids are widely established as immunosuppressants in LuTx patients [71]. It is currently acknowledged that severe forms of COVID-19 are associated with the release of cytokines, such as IL-2, IL-6, IL-7, IL-10, tumor necrosis factor (TNF), and granulocyte colony-stimulating factor (GCSF) [72]. Schoot et al. highlighted that corticosteroids mitigate the effects of the cytokine storm [57]. Lai et al. further demonstrated that SARS-CoV-2-positive transplanted patients treated with corticosteroids may display more intense and prolonged virus shedding [63]. According to Schoot et al., the effects of corticosteroids on viral replication remain to be clarified [57]. On the other hand, several studies indicate that corticosteroid discontinuation is not beneficial for LuTx patients [3,73].

Azitromycin is often administered to LuTx patients as a prophylaxis of chronic lung allograft dysfunction (CLAD) [74]. Even though the association with CLAD is not fully understood, the administration of azithromycin is associated with improved survival [75]. There are several studies supporting azithromycin administration in COVID-19-infected SOT patients [65,76,77].

Mammalian target of rapamycin (mTOR) inhibitors, such as sirolimus and everolimus, are increasingly used after lung transplantation [78]. To date, there are no specific data nor recommendations regarding their administration during COVID-19 infection.

Other studies did not identify an association between the intensity of immunosuppressive treatment and the clinical course of COVID-19 infection. Messika et al. did not find a correlation between recent intensification of immunosuppression and a poor prognosis of COVID-19 disease [79]. Moreover, in a study by Zaidan et al., the association between the intensity of baseline immunosuppression and the COVID-19 outcome in SOT was not observed [65]. According to Pereira et al., chronic immunosuppressive therapy does not necessarily accompany the poor prognosis of COVID-19-infected patients [77]. Furthermore, the authors reported that most transplant centers currently maintain calcineurin inhibitors and prednisone, whereas the increase of prednisone dosage or its substitution by dexamethasone should likely be discussed in hypoxemic patients [65,80].

Aversa et al. reported no association between higher doses of immunosuppression at baseline and the severity of the COVID-19 disease. However, patients receiving thymoglobulin, antibody-depleting therapy, and high-dose corticosteroids within the preceding three months were prone to develop severe disease [81].

The understanding of the pharmacological interactions between anti-COVID-19 drugs and immunosuppressive drugs is far from satisfactory. Dexamethasone was found to affect the plasma concentrations of sirolimus and tacrolimus [82]. Moreover, as tacrolimus is well-known for its nephrotoxicity, renal function should be evaluated before the administration of remdesivir, and further monitored [83]. Currently, clinical trials evaluating the in vivo drug interactions in COVID-19-infected SOT patients are lacking.

Questions about the use of systemic *immunosuppressive* agents have become a great concern during the COVID-19 pandemic. The outcomes of patients have been examined, and in the course of time, total discontinuation of the treatment was not recommended. Thus, the daily reality for many patients after lung transplantation, with regard to the use of immunosuppressive medication, has not changed during the pandemic. However, the psychological burden of immunosuppression during the COVID-19 pandemic may have been enormous for LuTx recipients, given the fact that SOT recipients were labeled amongst the most vulnerable subpopulations due to their immunosuppressive treatment.

## 7. SARS-CoV-2-Infected Recipient—COVID-19 Treatment

The treatment of COVID-19 in SOT recipients remains challenging [84]. Remdesivir has become a treatment of choice in LuTx centers, from the very beginning of the pandemic [9]. In certain subgroups of patients, remdesivir administration was found to promote fast recovery from COVID-19 [85,86,87]. Unfortunately, studies addressing remdesivir efficacy and safety in the LuTx population are currently missing.

Similarly, favipiravir, as an inhibitor of RNA polymerase, was also shown to enhance the viral clearance in a study by Manabe et al. [88]. Although the therapy with favipiravir was implemented in several LuTx centers, clinical trials supporting the role of favipiravir in LuTx patients were not yet initiated [88].

The clinical benefit of convalescent plasma (CP) in the treatment of COVID-19 is currently controversial [89,90]. The therapeutic potential of CP is being discussed particularly in immunosuppressed patients [91,92]. A study by Rahman et al. investigated the efficacy and safety of CP in thirteen SOT recipients [93]. In this study, eight patients experienced a significant clinical improvement, and none of the patients exhibited drug-related adverse reactions. To note, three patients died in the study cohort [93].

Sarrell et al. evaluated the efficacy and safety of monoclonal antibody (mAbs) therapy in SOT recipients [94]. The mAbs therapy was safe and associated with a lower risk of hospitalization [94]. Bamlanivimab monotherapy was shown in SOT recipients to be a well-tolerated option for the treatment of mild-to-moderate COVID-19 [95]. However, the efficacy of banlanivimab in combination with etesevimab has not yet been elucidated in SOT recipients [95].

The subcutaneous administration of casivirimab-imdevimab (REGEN-COV) was shown to prevent COVID-19 in previously uninfected household contacts in the general population, and, therefore, might be of importance in the prevention of COVID-19 [96]. A study by Dhand et al. evaluated 25 COVID-19-infected SOT recipients receiving REGEN-COV, and demonstrated neither COVID-19 progressions nor hospitalizations in the study participants [97]. Nevertheless, observational studies evaluating the pre-exposure prophylactic administration of mAbs in LuTx recipients are awaited.

Taken together, the majority of COVID-19-infected LuTx individuals can recover, but may require vigilant monitoring for secondary infections, and may decline in spirometric lung functions. Therapeutic management of the infected LuTx recipients may still challenge physicians, and to find the most feasible approach in different sub-groups of patients, more research is needed.

## 8. Conclusions

Lung transplantations became one of the most affected medical areas of the COVID-19 outbreak [5]. The immunosuppressive nature of post-transplant (anti-rejection) medication has labeled patients undergoing SOT as highly vulnerable, and, ultimately, novel transplantation policies had to be created in response to the increased risk of infection and death associated with COVID-19 [3,4,5]. In our study, we have closely reviewed the consequences of the COVID-19 pandemic on lung transplantation.

The impact of the COVID-19 pandemic on worldwide organ transplantation activity was evident in most countries. Though the US LuTx centers reported regular transplant activity in the early phase of the pandemic, in the UK, the overall LuTx activity significantly declined and led to a specific period of restricting lung transplantation to urgent cases [20,22,23]. These restrictions were further applied in most transplant centers, whereas several transplant centers also reported difficulties due to the shortage of ICU capacities [9,23].

Lungs from donors with ongoing SARS-CoV-2 infection could not be utilized, and, therefore, extensive screening for SARS-CoV-2 in both the upper and lower respiratory tract was recommended [15,28]. The sensitivity of RT-PCR testing was proven to be the highest in samples obtained from BAL [15].

COVID-19-associated ARDS newly became an indication for LuTx [38]. However, the most critical aspects, such as the selection criteria of possible transplant candidates and the optimal timing of surgery, remain to be answered [38,39,49].

High-dose corticosteroids, remdesivir, and monoclonal antibodies were the most commonly discussed treatment choices in LuTx recipients [3,9,73,94]. Calcineurin inhibitors were shown to block the SARS-CoV-2 replication, and together with MPA, are proposed as a possible treatment strategy for COVID-19 [57,62,63,67]. Corticosteroid discontinuation was not found to be beneficial for LuTx patients, as they mitigate the effects of the cytokine storm during COVID-19 infection [57]. In contrast, dose-tapering of MPA should be critically discussed in each patient [64,65,68,69]. Renal functions require monitoring in remdesivir-treated LuTx patients, as tacrolimus concentrations increase with SARS-CoV-2 infection [80,83].

## Figures and Tables

**Table 1:** COVID-19-associated ARDS recipient evaluation—major aspects to be considered in candidates for lung transplantation based on available data.

Factors to be Considered when Assessing a Patient with COVID-19-Associated ARDS as a Potential Candidate for Lung Transplantation	
1.	Potential candidates should be younger than 65 years, as ECMO bridge to lung transplantation demonstrated inferior outcomes in older patients [39,49].
2.	Potential candidates should not exhibit any other extrapulmonary organ failures, and should not have pre-existing unmanageable comorbidities [39,49].
3.	A sufficient period should elapse to provide adequate time for native lung recovery. Transplantation should not be considered less than 4–8 weeks after the initial clinical signs of respiratory failure. Transplantation should not be considered if an ongoing improvement is registered, regardless of the time elapsed [39,49].
4.	Radiological evidence of irreversible lung disease (such as bullous destruction, established interstitial fibrosis, traction, cystic bronchiectasis, extensive parenchymal consolidation, and hydropneumothorax) should be present. However, radiological findings alone should not be used to determine recoverability [38,39,49].
5.	The awake ECMO bridge to lung transplantation concept proved better outcomes compared to the non-awake ECMO concept [51]. Therefore, the potential candidate should preferably be awake on ECMO, and participate in physical rehabilitation while on a transplantation waiting list. Moreover, the ability to provide first-person consent, and understanding the impact of transplantation on quality of life before surgery is beneficial. If a patient is not awake, reactive, and physically active, an exception is possible in patients with a high potential for post-transplant recovery. However, in such cases, informed consent from next-of-kin or a reliable medical power of attorney should be obtained [38,39,49].
6.	Negative SARS-CoV-2 RT-PCR testing from the lower respiratory tract should be repeatedly confirmed. In patients separated from mechanical ventilation with no tracheostomy, repeated RT-PCR from the nasopharyngeal swab is proposed. Viral cultures can be used as well. Antibodies should also be evaluated before transplantation [38,39,49].
7.	Lung transplantation in patients bridged on ECMO for ARDS belongs to the most complex procedures in the field. Therefore, transplantation centers performing lung transplantation for COVID-19-associated ARDS should have considerable experience with such high-risk transplantation (Cypel M, 2020) [49].
8.	Ethical consideration is one of the major concerns in patients with COVID-19-associated ARDS bridged to lung transplantation. Therefore, the center should have access to a broad donor pool and low waiting-list mortality to be able to ensure an unbiased organ allocation [49].
9.	The potential candidate’s medical condition and transplantability should be critically re-evaluated periodically by a multidisciplinary team [38].
10.	Only double-lung transplantation should be considered. This is the best option due to often-seen underlying pulmonary hypertension and superimposed nosocomial infections, which might lead to severe pneumonia after receiving post-transplantation immunosuppression [39].

## Data Availability

Not applicable.

## Abbreviations

anti-IL-6	Anti-interleukin-6
ARDS	Acute respiratory distress syndrome
AST	American Society of Transplantation
BAL	Bronchoalveolar lavage
bDMARDs	Biological disease-modifying antirheumatic drugs
CD4+	Cluster of differentiation 4 positive
CD8+	Cluster of differentiation 8 positive
CLAD	Chronic lung allograft dysfunction
CNIs	Calcineurin inhibitors
COVID-19	Coronavirus disease 2019
CP	Convalescent plasma
CsA	Cyclosporin A
CT	Computed tomography
Ct	Cycle threshold
ECMO	Extracorporeal membrane oxygenation
GCSF	Granulocyte colony-stimulating factor
ICU	Intensive care unit
IgG	Immunoglobulin G
IL-2	Interleukin-2
IL-6	Interleukin-6
IL-7	Interleukin-7
IL-10	Interleukin-10
ISHLT	International Society for Heart and Lung Transplantation
LuTx	Lung transplant
mAbs	monoclonal antibodies
MPA	Mycophenolic acid
mRNA	Messenger RNA
mTOR	Mammalian target of rapamycin
NFAT	Nuclear factor of activated T cells
NP	Nasopharyngeal
PCR	Polymerase chain reaction
RNA	Ribonucleic acid
RT-PCR	Real-time polymerase chain reaction
SOT	Solid organ transplantation
SARS-CoV-2	Severe acute respiratory syndrome coronavirus 2
TNF	Tumor necrosis factor
Tx	Transplant
UK	United Kingdom
US	United States of America
VA-ECMO	Venoarterial extracorporeal membrane oxygenation
VV-ECMO	Venovenous extracorporeal membrane oxygenation
WHO	World Health Organization
